# (±)-4,12,15,18,26-Penta­hydroxy-13,17-dioxahepta­cyclo­[14.10.0.0^3,14^.0^4,12^.0^6,11^.0^18,26^.0^19,24^]hexa­cosa-1,3(14),6(11),7,9,15,19,21,23-nona­ene-5,25-dione monohydrate

**DOI:** 10.1107/S1600536811009573

**Published:** 2011-03-19

**Authors:** Khalid Mahmood, Muhammad Yaqub, M. Nawaz Tahir, Zahid Shafiq, Ashfaq Mahmood Qureshi

**Affiliations:** a Department of Chemistry, Bahauddin Zakariya University, Multan 60800, Pakistan; b Department of Physics, University of Sargodha, Sargodha, Pakistan

## Abstract

The title compound, C_24_H_14_O_9_·H_2_O, displays a cup-shaped form. The water mol­ecule is disordered over two set of sites with an occupancy ratio of 0.78:0.22. The mol­ecule of the compound has four stereocenters and corresponds to the *SSRR*/*RRSS* diastereoisomer. In the mol­ecule, the maximum dihedral angle between the planar benzene rings is 80.40 (4)°. The H atoms of the hy­droxy groups are engaged in hydrogen bonding, forming infinite chains parallel to the *a* axis. These chains are inter­linked through water mol­ecules, resulting in the formation of a two-dimensional network parallel to the (001) plane. Futhermore C—H⋯O, C—H⋯π and slipped π–π inter­actions result in the formation of a three-dimensional network.

## Related literature

For background and related structures, see: Almog *et al.* (2009 ▶); Yaqub *et al.* (2010 ▶).
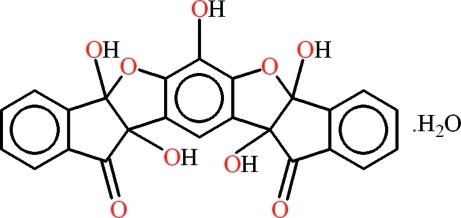


## Experimental

### 

#### Crystal data

C_24_H_14_O_9_·H_2_O
*M* *_r_* = 464.37
Triclinic, 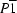

*a* = 8.2448 (4) Å
*b* = 11.1558 (7) Å
*c* = 12.2569 (7) Å
α = 64.571 (2)°
β = 78.126 (1)°
γ = 80.738 (2)°
*V* = 992.98 (10) Å^3^
*Z* = 2
Mo *K*α radiation
μ = 0.12 mm^−1^
*T* = 296 K
0.24 × 0.16 × 0.14 mm

#### Data collection

Bruker Kappa APEXII CCD diffractometer
Absorption correction: multi-scan (*SADABS*; Bruker, 2005 ▶) *T*_min_ = 0.975, *T*_max_ = 0.983
14323 measured reflections
3592 independent reflections
2717 reflections with *I* > 2σ(*I*)
*R*_int_ = 0.039

#### Refinement

*R*[*F* ^2^ > 2σ(*F* ^2^)] = 0.041
*wR*(*F* ^2^) = 0.106
*S* = 1.03
3592 reflections
312 parameters
H-atom parameters constrained
Δρ_max_ = 0.29 e Å^−3^
Δρ_min_ = −0.22 e Å^−3^

### 

Data collection: *APEX2* (Bruker, 2009 ▶); cell refinement: *SAINT* (Bruker, 2009 ▶); data reduction: *SAINT*; program(s) used to solve structure: *SHELXS97* (Sheldrick, 2008 ▶); program(s) used to refine structure: *SHELXL97* (Sheldrick, 2008 ▶); molecular graphics: *ORTEPIII* (Burnett & Johnson, 1996 ▶), *ORTEP-3 for Windows* (Farrugia, 1997 ▶) and *PLATON* (Spek, 2009 ▶); software used to prepare material for publication: *WinGX* (Farrugia, 1999 ▶) and *PLATON*.

## Supplementary Material

Crystal structure: contains datablocks global, I. DOI: 10.1107/S1600536811009573/dn2665sup1.cif

Structure factors: contains datablocks I. DOI: 10.1107/S1600536811009573/dn2665Isup2.hkl

Additional supplementary materials:  crystallographic information; 3D view; checkCIF report

## Figures and Tables

**Table 1:** Hydrogen-bond geometry (Å, °) *Cg*1 is the centroid of the C1–C6 ring.

*D*—H⋯*A*	*D*—H	H⋯*A*	*D*⋯*A*	*D*—H⋯*A*
O1—H1⋯O4^i^	0.82	2.00	2.6885 (18)	141
O3—H3⋯O8^ii^	0.82	1.94	2.7559 (19)	177
O4—H4⋯O6^ii^	0.82	2.08	2.8964 (18)	176
O7—H7⋯O2^ii^	0.82	2.14	2.9548 (19)	175
O8—H8⋯O10*A*	0.82	1.86	2.653 (3)	163
O8—H8⋯O10*B*	0.82	1.91	2.587 (3)	139
O10*A*—H10*A*⋯O9^i^	0.85	2.01	2.844 (3)	168
O10*B*—H10*C*⋯O1^iii^	0.96	2.50	3.357 (5)	148
C11—H11⋯O9^iv^	0.93	2.44	3.345 (3)	164
C23—H23⋯*Cg*1^v^	0.93	2.65	3.562 (2)	168

Symmetry codes: (i) 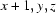; (ii) 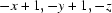; (iii) 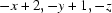; (iv) 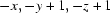; (v) 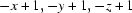.

**Table 2:** Table 2 ▶ π-π stacking inter­actions (Å,°) *Cg*1, *Cg*2 and *Cg*3 are the centroids of the C1–C6, C10–C15 and C19–C24 rings, respectively.

*CgI*	*CgJ*	*CgI*⋯*CgJ*^*a*^	*CgI*⋯*P(J)*^*b*^	*CgJ*⋯*P(I)*^*c*^	Slippage
*Cg*1	*Cg*1^vi^	3.5314 (11)	3.396	3.396	0.968
*Cg*2	*Cg*2^vii^	3.6525 (14)	3.377	3.377	1.392
*Cg*3	*Cg*3^v^	3.7905 (14)	3.358	3.358	1.758

Symmetry codes: (v) 1 − *x*, 1 − *y*, 1 − *z*; (vi) 1 − *x*, 1 − *y*, −*z*; (vii) −*x*, −*y*, 1 − *z*. Notes: (*a*) distance between centroids; (*b*) perpendicular distance of *CgI* on ring plan *J*; (*c*) perpendicular distance of *CgJ* on ring plan *I*; (*d*) slippage = vertical displacement between ring centroids.

**Fig. 1.**
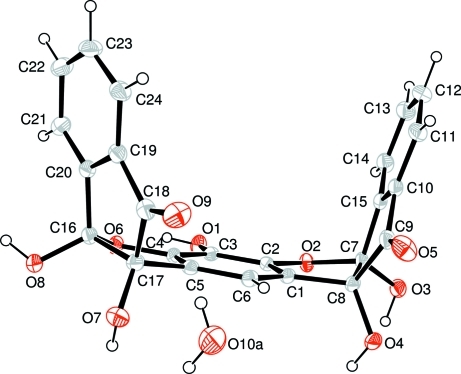
View of the title compound with the atom numbering scheme. The thermal ellipsoids are drawn at the 30% probability level. H-atoms are shown by small circles of arbitrary radii. For the sake of clarity, only the major component of the disordered water molecule is represented.

**Fig. 2.**
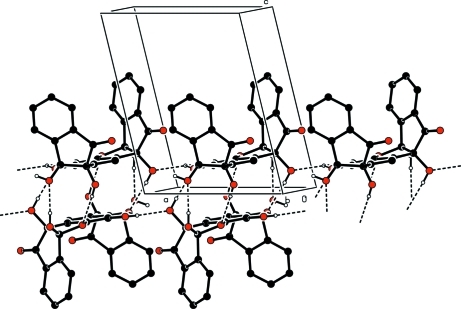
Partial packing view showing the formation of chain parallel to the a axis. For clarity H atoms not involved in hydrogen bondings and disordered water molecule have been omitted. H bonds are represented as dashed line.

## supplementary crystallographic information

### Comment

We have recently reported the crystal structure of 6,7,8,9-tetrahydro-4 b,9 b-dihydroxyindano[1,2-*b*]indoline-9,10-dione monohydrate (Yaqub *et al.*, 2010) synthesized from ninhydrine. In continuation to the synthesis of biologically important ninhydrine derivatives, we report herein the structure and preparation of the title compound (I).

The title compound (I) displays a cup shaped form (Fig. 1). In (I), the central group A (C1–C6/C8/C17/O1/O2/O6), the armed groups B (C7/C9/O5/C10—C15) and C (C16/C18/O9/C19–C24) are planar with r. m. s. deviations of 0.0250, 0.0462 and 0.0190 Å, respectively. The adjacent atoms C7 and C16 to central group A are at a distance of 0.3706 (22) and 0.3424 (22) Å, respectively from the mean square plane and thus forming envelop form from two sides. The group B also form envelop shape with C8 at a distance of 0.3203 (23) Å from its mean square plane. The C17 atom is at a distance of -0.1997 (24) Å from the mean square plane of group C. The dihedral angle between A/B, A/C and B/C is 80.40 (4)°, 78.55 (4)° and 38.59 (4)°, respectively. The molecule of (I) has stereo centers at C7, C8, C16 and C17 and corresponds to the SSRR/RRSS diastereoisomer. Intermolecular O—H···O hydrogen bonds involving the hydroxy groups build up infinite one dimensional chain parallel to the a axis (Table 1, Fig. 2) . Hydrogen bonds involving the water molecule link the chains to form a two dimensionnal network parallel to the (0 0 1) plane (Table 1) and weak C-H···O hydrogen bonds (Table 1) connect the sheet to build up a three dimensional network. The packing is further stabilized through C-H···π and slippest π—π interactions (Tables 1 and 2).

The crystal structure of (I) is closeled related to the structure of 4b,7a,12*a*,13,13*b*-pentahydroxy-4 b,7a,12*a*, 13*b*-tetrahydro-12H,14*H*-indeno[1,2-*b*]indeno[2',1':4,5]furo[3,2-*f*][1] benzofuran-12,14-dione methanol solvate (Almog *et al.*, 2009). The differences with I are due to the bonding around phenol ring and inclusion of methanol solvate instead of water.

### Experimental

The pyrogallol (0.10 g, 0.793 mmol) was added to the stirred solution of ninhydrin (0.29 g, 1.586 mmol) in 15 ml of acetic acid at 323 K for 45 min and kept at room temperature for five days in a closed vessel. White colorless prisms for *x*-ray analysis of the title compound (I) were separated and washed with acetic acid and Petrolium ether.

Yield: 0.29 g, 74% m.p. 548 K

### Refinement

The high values of thermal parameters of the solvent water lead to the disorder. The water molecule is disordered over two set of sites with occupancy ratio of groups is 0.786 (5):0.214 (5). The disordered water molecules were treated with equal thermal parameters. One of the H-atom shares both disordered molecules.

All H atoms attached to C atoms and O atom of hydroxy groups were fixed geometrically and treated as riding with C—H = 0.93 and O—H = 0.82 Å with U_iso_(H) = 1.2U_eq_(C) or U_iso_(H) = 1.5U_eq_(O). H atoms of the disordered water molecule were located in difference Fourier maps and included in the subsequent refinement using restraints (O-H = 0.85 (1)Å and H···H = 1.40 (2)Å) with U_iso_(H) = 1.5U_eq_(O). In the last cycles of refinement they were treated as riding on their parent O atom.

### Crystal data

**Table:**

C_24_H_14_O_9_·H_2_O	*Z* = 2
*M**_r_* = 464.37	*F*(000) = 480
Triclinic, *P*1	*D*_x_ = 1.553 Mg m^−^^3^
Hall symbol: -P 1	Mo *K*α radiation, λ = 0.71073 Å
*a* = 8.2448 (4) Å	Cell parameters from 2717 reflections
*b* = 11.1558 (7) Å	θ = 2.1–25.3°
*c* = 12.2569 (7) Å	µ = 0.12 mm^−^^1^
α = 64.571 (2)°	*T* = 296 K
β = 78.126 (1)°	Prisms, white
γ = 80.738 (2)°	0.24 × 0.16 × 0.14 mm
*V* = 992.98 (10) Å^3^	

### Data collection

**Table:**

Bruker Kappa APEXII CCD diffractometer	3592 independent reflections
Radiation source: fine-focus sealed tube	2717 reflections with *I* > 2σ(*I*)
graphite	*R*_int_ = 0.039
Detector resolution: 8.10 pixels mm^-1^	θ_max_ = 25.3°, θ_min_ = 2.1°
ω scans	*h* = −9→9
Absorption correction: multi-scan (*SADABS*; Bruker, 2005)	*k* = −13→13
*T*_min_ = 0.975, *T*_max_ = 0.983	*l* = −14→14
14323 measured reflections	

### Refinement

**Table:**

Refinement on *F*^2^	Primary atom site location: structure-invariant direct methods
Least-squares matrix: full	Secondary atom site location: difference Fourier map
*R*[*F*^2^ > 2σ(*F*^2^)] = 0.041	Hydrogen site location: inferred from neighbouring sites
*wR*(*F*^2^) = 0.106	H-atom parameters constrained
*S* = 1.03	*w* = 1/[σ^2^(*F*_o_^2^) + (0.0477*P*)^2^ + 0.3139*P*] where *P* = (*F*_o_^2^ + 2*F*_c_^2^)/3
3592 reflections	(Δ/σ)_max_ < 0.001
312 parameters	Δρ_max_ = 0.29 e Å^−^^3^
0 restraints	Δρ_min_ = −0.22 e Å^−^^3^

### Special details

**Table:**

Geometry. All esds (except the esd in the dihedral angle between two l.s. planes) are estimated using the full covariance matrix. The cell esds are taken into account individually in the estimation of esds in distances, angles and torsion angles; correlations between esds in cell parameters are only used when they are defined by crystal symmetry. An approximate (isotropic) treatment of cell esds is used for estimating esds involving l.s. planes.
Refinement. Refinement of *F*^2^ against ALL reflections. The weighted *R*-factor *wR* and goodness of fit *S* are based on *F*^2^, conventional *R*-factors *R* are based on *F*, with *F* set to zero for negative *F*^2^. The threshold expression of *F*^2^ > σ(*F*^2^) is used only for calculating *R*-factors(gt) *etc*. and is not relevant to the choice of reflections for refinement. *R*-factors based on *F*^2^ are statistically about twice as large as those based on *F*, and *R*- factors based on ALL data will be even larger.

### Fractional atomic coordinates and isotropic or equivalent isotropic displacement parameters (Å^2^)

**Table:**

	*x*	*y*	*z*	*U*_iso_*/*U*_eq_	Occ. (<1)
O1	0.74158 (16)	0.31117 (14)	0.13753 (13)	0.0325 (4)	
H1	0.8190	0.3518	0.1328	0.049*	
O2	0.44555 (15)	0.20020 (13)	0.18069 (12)	0.0275 (3)	
O3	0.23840 (18)	0.07962 (14)	0.18599 (13)	0.0345 (4)	
H3	0.2547	0.1146	0.1110	0.052*	
O4	0.06749 (16)	0.34145 (16)	0.10489 (12)	0.0340 (4)	
H4	0.1297	0.3594	0.0395	0.051*	
O5	−0.08231 (19)	0.34995 (16)	0.32765 (14)	0.0470 (4)	
O6	0.70474 (15)	0.58630 (14)	0.12331 (12)	0.0278 (3)	
O7	0.38306 (18)	0.82777 (14)	0.04490 (13)	0.0367 (4)	
H7	0.4280	0.8243	−0.0200	0.055*	
O8	0.71372 (18)	0.81081 (15)	0.06590 (12)	0.0348 (4)	
H8	0.7947	0.8166	0.0920	0.052*	
O9	0.20667 (19)	0.75200 (17)	0.28466 (15)	0.0472 (4)	
O10A	0.9905 (3)	0.8676 (3)	0.1058 (3)	0.1166 (15)	0.78
H10A	1.0445	0.8386	0.1654	0.175*	0.78
H10B	1.0535	0.9036	0.0381	0.175*	
O10B	1.0286 (3)	0.8307 (3)	0.0423 (3)	0.1166 (15)	0.22
H10C	1.0828	0.8252	−0.0326	0.175*	0.22
C1	0.2922 (2)	0.3946 (2)	0.17679 (16)	0.0237 (4)	
C2	0.4482 (2)	0.32831 (19)	0.16793 (16)	0.0229 (4)	
C3	0.5959 (2)	0.38453 (19)	0.14809 (16)	0.0229 (4)	
C4	0.5743 (2)	0.5138 (2)	0.13981 (16)	0.0236 (4)	
C5	0.4192 (2)	0.58264 (19)	0.14865 (16)	0.0239 (4)	
C6	0.2742 (2)	0.5243 (2)	0.16695 (17)	0.0254 (4)	
H6	0.1701	0.5699	0.1724	0.031*	
C7	0.2716 (2)	0.1651 (2)	0.23039 (17)	0.0267 (5)	
C8	0.1629 (2)	0.3014 (2)	0.20018 (17)	0.0257 (4)	
C9	0.0451 (2)	0.2811 (2)	0.32021 (18)	0.0292 (5)	
C10	0.1173 (2)	0.1670 (2)	0.41783 (18)	0.0284 (5)	
C11	0.0688 (3)	0.1197 (2)	0.54396 (19)	0.0365 (5)	
H11	−0.0191	0.1630	0.5777	0.044*	
C12	0.1552 (3)	0.0069 (2)	0.6172 (2)	0.0424 (6)	
H12	0.1258	−0.0260	0.7017	0.051*	
C13	0.2853 (3)	−0.0581 (2)	0.5666 (2)	0.0415 (6)	
H13	0.3413	−0.1343	0.6179	0.050*	
C14	0.3335 (3)	−0.0120 (2)	0.44149 (19)	0.0350 (5)	
H14	0.4213	−0.0556	0.4080	0.042*	
C15	0.2470 (2)	0.1011 (2)	0.36760 (18)	0.0268 (5)	
C16	0.6316 (2)	0.7026 (2)	0.15031 (17)	0.0277 (5)	
C17	0.4423 (2)	0.7165 (2)	0.14245 (17)	0.0276 (5)	
C18	0.3551 (3)	0.7255 (2)	0.26280 (19)	0.0311 (5)	
C19	0.4801 (3)	0.6899 (2)	0.34347 (18)	0.0303 (5)	
C20	0.6377 (2)	0.6727 (2)	0.28187 (18)	0.0284 (5)	
C21	0.7748 (3)	0.6349 (2)	0.34150 (19)	0.0359 (5)	
H21	0.8802	0.6208	0.3018	0.043*	
C22	0.7509 (3)	0.6189 (2)	0.4618 (2)	0.0426 (6)	
H22	0.8420	0.5950	0.5029	0.051*	
C23	0.5942 (3)	0.6377 (2)	0.5225 (2)	0.0426 (6)	
H23	0.5821	0.6272	0.6031	0.051*	
C24	0.4564 (3)	0.6717 (2)	0.46534 (19)	0.0373 (5)	
H24	0.3508	0.6822	0.5066	0.045*	

### Atomic displacement parameters (Å^2^)

**Table:**

	*U*^11^	*U*^22^	*U*^33^	*U*^12^	*U*^13^	*U*^23^
O1	0.0166 (7)	0.0386 (9)	0.0433 (9)	−0.0027 (6)	−0.0024 (6)	−0.0186 (7)
O2	0.0203 (7)	0.0296 (8)	0.0319 (8)	−0.0061 (6)	0.0026 (6)	−0.0135 (6)
O3	0.0396 (9)	0.0396 (9)	0.0299 (8)	−0.0151 (7)	0.0011 (7)	−0.0181 (7)
O4	0.0214 (8)	0.0528 (10)	0.0270 (8)	−0.0106 (7)	−0.0038 (6)	−0.0130 (7)
O5	0.0327 (9)	0.0492 (10)	0.0442 (10)	0.0055 (8)	0.0066 (7)	−0.0139 (8)
O6	0.0220 (7)	0.0351 (8)	0.0299 (7)	−0.0086 (6)	0.0005 (6)	−0.0165 (6)
O7	0.0418 (9)	0.0335 (9)	0.0308 (8)	−0.0006 (7)	−0.0062 (7)	−0.0100 (7)
O8	0.0383 (9)	0.0398 (9)	0.0264 (8)	−0.0185 (7)	−0.0032 (6)	−0.0093 (7)
O9	0.0341 (10)	0.0651 (12)	0.0484 (10)	0.0044 (8)	−0.0016 (7)	−0.0338 (9)
O10A	0.102 (2)	0.136 (3)	0.081 (2)	−0.074 (2)	−0.0554 (19)	0.0243 (19)
O10B	0.102 (2)	0.136 (3)	0.081 (2)	−0.074 (2)	−0.0554 (19)	0.0243 (19)
C1	0.0183 (10)	0.0325 (11)	0.0206 (10)	−0.0043 (8)	−0.0019 (7)	−0.0109 (9)
C2	0.0233 (11)	0.0270 (11)	0.0178 (9)	−0.0056 (8)	−0.0005 (7)	−0.0086 (8)
C3	0.0178 (10)	0.0309 (11)	0.0190 (9)	−0.0020 (8)	−0.0016 (7)	−0.0096 (8)
C4	0.0214 (11)	0.0318 (11)	0.0186 (9)	−0.0094 (8)	−0.0002 (7)	−0.0101 (8)
C5	0.0243 (11)	0.0285 (11)	0.0183 (9)	−0.0033 (8)	−0.0021 (7)	−0.0093 (8)
C6	0.0181 (10)	0.0332 (12)	0.0242 (10)	−0.0003 (8)	−0.0025 (8)	−0.0119 (9)
C7	0.0204 (11)	0.0331 (12)	0.0281 (11)	−0.0104 (8)	0.0020 (8)	−0.0137 (9)
C8	0.0171 (10)	0.0362 (12)	0.0243 (10)	−0.0056 (8)	−0.0024 (8)	−0.0119 (9)
C9	0.0224 (11)	0.0352 (12)	0.0318 (11)	−0.0085 (9)	0.0017 (8)	−0.0157 (10)
C10	0.0247 (11)	0.0343 (12)	0.0281 (11)	−0.0103 (9)	0.0002 (8)	−0.0138 (9)
C11	0.0360 (13)	0.0461 (14)	0.0293 (12)	−0.0126 (10)	0.0046 (9)	−0.0183 (11)
C12	0.0475 (15)	0.0511 (15)	0.0250 (11)	−0.0179 (12)	−0.0023 (10)	−0.0089 (11)
C13	0.0397 (14)	0.0397 (14)	0.0356 (13)	−0.0072 (11)	−0.0113 (10)	−0.0026 (11)
C14	0.0300 (12)	0.0332 (12)	0.0378 (12)	−0.0044 (9)	−0.0036 (9)	−0.0109 (10)
C15	0.0237 (11)	0.0306 (11)	0.0283 (11)	−0.0096 (9)	−0.0017 (8)	−0.0124 (9)
C16	0.0290 (11)	0.0300 (11)	0.0250 (10)	−0.0093 (9)	0.0001 (8)	−0.0116 (9)
C17	0.0273 (11)	0.0312 (11)	0.0244 (10)	−0.0035 (9)	−0.0040 (8)	−0.0111 (9)
C18	0.0323 (13)	0.0288 (12)	0.0329 (12)	−0.0048 (9)	0.0014 (9)	−0.0153 (10)
C19	0.0370 (13)	0.0271 (11)	0.0287 (11)	−0.0064 (9)	−0.0005 (9)	−0.0139 (9)
C20	0.0343 (12)	0.0262 (11)	0.0265 (10)	−0.0077 (9)	−0.0028 (9)	−0.0116 (9)
C21	0.0361 (13)	0.0406 (13)	0.0349 (12)	−0.0059 (10)	−0.0078 (10)	−0.0172 (10)
C22	0.0551 (16)	0.0412 (14)	0.0367 (13)	−0.0050 (11)	−0.0176 (11)	−0.0159 (11)
C23	0.0667 (17)	0.0392 (14)	0.0255 (11)	−0.0086 (12)	−0.0077 (11)	−0.0150 (10)
C24	0.0502 (15)	0.0344 (13)	0.0287 (12)	−0.0090 (10)	0.0028 (10)	−0.0161 (10)

### Geometric parameters (Å, °)

**Table:**

O1—C3	1.356 (2)	C7—C15	1.501 (3)
O1—H1	0.8200	C7—C8	1.571 (3)
O2—C2	1.373 (2)	C8—C9	1.535 (3)
O2—C7	1.482 (2)	C9—C10	1.465 (3)
O3—C7	1.369 (2)	C10—C15	1.388 (3)
O3—H3	0.8200	C10—C11	1.393 (3)
O4—C8	1.414 (2)	C11—C12	1.379 (3)
O4—H4	0.8200	C11—H11	0.9300
O5—C9	1.210 (2)	C12—C13	1.387 (3)
O6—C4	1.379 (2)	C12—H12	0.9300
O6—C16	1.481 (2)	C13—C14	1.383 (3)
O7—C17	1.407 (2)	C13—H13	0.9300
O7—H7	0.8200	C14—C15	1.384 (3)
O8—C16	1.374 (2)	C14—H14	0.9300
O8—H8	0.8200	C16—C20	1.510 (3)
O9—C18	1.214 (2)	C16—C17	1.562 (3)
O10A—H10A	0.8488	C17—C18	1.540 (3)
O10A—H10B	0.8501	C18—C19	1.466 (3)
O10B—H10B	0.8488	C19—C20	1.391 (3)
O10B—H10C	0.9573	C19—C24	1.394 (3)
C1—C2	1.385 (3)	C20—C21	1.383 (3)
C1—C6	1.385 (3)	C21—C22	1.382 (3)
C1—C8	1.507 (3)	C21—H21	0.9300
C2—C3	1.389 (3)	C22—C23	1.385 (3)
C3—C4	1.386 (3)	C22—H22	0.9300
C4—C5	1.391 (3)	C23—C24	1.373 (3)
C5—C6	1.385 (3)	C23—H23	0.9300
C5—C17	1.503 (3)	C24—H24	0.9300
C6—H6	0.9300		
			
C3—O1—H1	109.5	C10—C11—H11	121.0
C2—O2—C7	106.21 (13)	C11—C12—C13	120.9 (2)
C7—O3—H3	109.5	C11—C12—H12	119.5
C8—O4—H4	109.5	C13—C12—H12	119.5
C4—O6—C16	106.53 (14)	C14—C13—C12	121.4 (2)
C17—O7—H7	109.5	C14—C13—H13	119.3
C16—O8—H8	109.5	C12—C13—H13	119.3
H10A—O10A—H10B	111.1	C13—C14—C15	117.9 (2)
H10B—O10B—H10C	105.5	C13—C14—H14	121.0
C2—C1—C6	120.97 (17)	C15—C14—H14	121.0
C2—C1—C8	108.59 (17)	C14—C15—C10	120.92 (19)
C6—C1—C8	130.42 (17)	C14—C15—C7	127.30 (18)
O2—C2—C1	114.10 (16)	C10—C15—C7	111.75 (18)
O2—C2—C3	122.06 (17)	O8—C16—O6	107.42 (15)
C1—C2—C3	123.84 (18)	O8—C16—C20	114.67 (16)
O1—C3—C4	127.48 (17)	O6—C16—C20	110.07 (16)
O1—C3—C2	118.53 (17)	O8—C16—C17	113.52 (16)
C4—C3—C2	113.99 (17)	O6—C16—C17	106.15 (14)
O6—C4—C3	123.20 (17)	C20—C16—C17	104.69 (15)
O6—C4—C5	113.39 (17)	O7—C17—C5	115.96 (16)
C3—C4—C5	123.41 (17)	O7—C17—C18	108.19 (16)
C6—C5—C4	121.19 (19)	C5—C17—C18	108.73 (15)
C6—C5—C17	129.73 (18)	O7—C17—C16	117.31 (16)
C4—C5—C17	108.98 (16)	C5—C17—C16	101.66 (15)
C5—C6—C1	116.59 (18)	C18—C17—C16	104.13 (15)
C5—C6—H6	121.7	O9—C18—C19	128.14 (19)
C1—C6—H6	121.7	O9—C18—C17	123.76 (19)
O3—C7—O2	108.40 (14)	C19—C18—C17	108.01 (17)
O3—C7—C15	110.20 (16)	C20—C19—C24	121.3 (2)
O2—C7—C15	110.56 (15)	C20—C19—C18	110.23 (18)
O3—C7—C8	118.18 (16)	C24—C19—C18	128.50 (19)
O2—C7—C8	105.64 (14)	C21—C20—C19	120.31 (19)
C15—C7—C8	103.66 (15)	C21—C20—C16	128.44 (18)
O4—C8—C1	114.40 (16)	C19—C20—C16	111.24 (18)
O4—C8—C9	108.96 (15)	C22—C21—C20	118.1 (2)
C1—C8—C9	111.02 (15)	C22—C21—H21	120.9
O4—C8—C7	116.81 (16)	C20—C21—H21	120.9
C1—C8—C7	101.22 (14)	C21—C22—C23	121.4 (2)
C9—C8—C7	103.80 (15)	C21—C22—H22	119.3
O5—C9—C10	128.69 (18)	C23—C22—H22	119.3
O5—C9—C8	124.02 (19)	C24—C23—C22	121.0 (2)
C10—C9—C8	107.30 (17)	C24—C23—H23	119.5
C15—C10—C11	120.9 (2)	C22—C23—H23	119.5
C15—C10—C9	109.91 (17)	C23—C24—C19	117.8 (2)
C11—C10—C9	129.16 (19)	C23—C24—H24	121.1
C12—C11—C10	118.0 (2)	C19—C24—H24	121.1
C12—C11—H11	121.0		
			
C7—O2—C2—C1	−13.7 (2)	C13—C14—C15—C10	0.8 (3)
C7—O2—C2—C3	166.22 (16)	C13—C14—C15—C7	−176.90 (19)
C6—C1—C2—O2	179.41 (16)	C11—C10—C15—C14	−1.0 (3)
C8—C1—C2—O2	0.8 (2)	C9—C10—C15—C14	−178.79 (17)
C6—C1—C2—C3	−0.5 (3)	C11—C10—C15—C7	176.95 (17)
C8—C1—C2—C3	−179.17 (17)	C9—C10—C15—C7	−0.8 (2)
O2—C2—C3—O1	2.0 (3)	O3—C7—C15—C14	62.6 (2)
C1—C2—C3—O1	−178.11 (16)	O2—C7—C15—C14	−57.2 (3)
O2—C2—C3—C4	−178.83 (16)	C8—C7—C15—C14	−170.02 (19)
C1—C2—C3—C4	1.1 (3)	O3—C7—C15—C10	−115.25 (18)
C16—O6—C4—C3	−166.01 (17)	O2—C7—C15—C10	124.93 (17)
C16—O6—C4—C5	13.3 (2)	C8—C7—C15—C10	12.1 (2)
O1—C3—C4—O6	−2.5 (3)	C4—O6—C16—O8	−140.03 (15)
C2—C3—C4—O6	178.36 (16)	C4—O6—C16—C20	94.49 (17)
O1—C3—C4—C5	178.20 (17)	C4—O6—C16—C17	−18.27 (18)
C2—C3—C4—C5	−0.9 (3)	C6—C5—C17—O7	−64.0 (3)
O6—C4—C5—C6	−179.17 (16)	C4—C5—C17—O7	119.56 (18)
C3—C4—C5—C6	0.2 (3)	C6—C5—C17—C18	58.1 (3)
O6—C4—C5—C17	−2.4 (2)	C4—C5—C17—C18	−118.33 (17)
C3—C4—C5—C17	176.96 (17)	C6—C5—C17—C16	167.58 (19)
C4—C5—C6—C1	0.5 (3)	C4—C5—C17—C16	−8.87 (19)
C17—C5—C6—C1	−175.59 (18)	O8—C16—C17—O7	6.5 (2)
C2—C1—C6—C5	−0.3 (3)	O6—C16—C17—O7	−111.32 (18)
C8—C1—C6—C5	178.02 (18)	C20—C16—C17—O7	132.24 (17)
C2—O2—C7—O3	147.83 (15)	O8—C16—C17—C5	134.01 (16)
C2—O2—C7—C15	−91.28 (18)	O6—C16—C17—C5	16.24 (17)
C2—O2—C7—C8	20.25 (17)	C20—C16—C17—C5	−100.20 (16)
C2—C1—C8—O4	−114.96 (18)	O8—C16—C17—C18	−113.04 (17)
C6—C1—C8—O4	66.6 (3)	O6—C16—C17—C18	129.19 (15)
C2—C1—C8—C9	121.23 (17)	C20—C16—C17—C18	12.75 (19)
C6—C1—C8—C9	−57.3 (3)	O7—C17—C18—O9	45.9 (3)
C2—C1—C8—C7	11.54 (19)	C5—C17—C18—O9	−80.8 (2)
C6—C1—C8—C7	−166.95 (19)	C16—C17—C18—O9	171.4 (2)
O3—C7—C8—O4	−15.6 (2)	O7—C17—C18—C19	−137.08 (16)
O2—C7—C8—O4	105.87 (17)	C5—C17—C18—C19	96.20 (18)
C15—C7—C8—O4	−137.81 (16)	C16—C17—C18—C19	−11.6 (2)
O3—C7—C8—C1	−140.47 (16)	O9—C18—C19—C20	−177.4 (2)
O2—C7—C8—C1	−19.02 (17)	C17—C18—C19—C20	5.8 (2)
C15—C7—C8—C1	97.30 (16)	O9—C18—C19—C24	3.6 (4)
O3—C7—C8—C9	104.36 (18)	C17—C18—C19—C24	−173.2 (2)
O2—C7—C8—C9	−134.19 (14)	C24—C19—C20—C21	0.9 (3)
C15—C7—C8—C9	−17.87 (18)	C18—C19—C20—C21	−178.18 (18)
O4—C8—C9—O5	−37.0 (3)	C24—C19—C20—C16	−178.04 (18)
C1—C8—C9—O5	89.9 (2)	C18—C19—C20—C16	2.9 (2)
C7—C8—C9—O5	−162.1 (2)	O8—C16—C20—C21	−63.9 (3)
O4—C8—C9—C10	143.22 (16)	O6—C16—C20—C21	57.4 (3)
C1—C8—C9—C10	−89.92 (19)	C17—C16—C20—C21	171.1 (2)
C7—C8—C9—C10	18.09 (19)	O8—C16—C20—C19	114.97 (19)
O5—C9—C10—C15	168.8 (2)	O6—C16—C20—C19	−123.79 (17)
C8—C9—C10—C15	−11.4 (2)	C17—C16—C20—C19	−10.1 (2)
O5—C9—C10—C11	−8.7 (4)	C19—C20—C21—C22	−1.8 (3)
C8—C9—C10—C11	171.1 (2)	C16—C20—C21—C22	176.9 (2)
C15—C10—C11—C12	1.0 (3)	C20—C21—C22—C23	1.0 (3)
C9—C10—C11—C12	178.2 (2)	C21—C22—C23—C24	0.7 (4)
C10—C11—C12—C13	−0.6 (3)	C22—C23—C24—C19	−1.6 (3)
C11—C12—C13—C14	0.4 (3)	C20—C19—C24—C23	0.8 (3)
C12—C13—C14—C15	−0.4 (3)	C18—C19—C24—C23	179.7 (2)

### Hydrogen-bond geometry (Å, °)

**Table:**

*D*—H···*A*	*D*—H	H···*A*	*D*···*A*	*D*—H···*A*
O1—H1···O4^i^	0.82	2.00	2.6885 (18)	141
O3—H3···O8^ii^	0.82	1.94	2.7559 (19)	177
O4—H4···O6^ii^	0.82	2.08	2.8964 (18)	176
O7—H7···O2^ii^	0.82	2.14	2.9548 (19)	175
O8—H8···O10A	0.82	1.86	2.653 (3)	163
O8—H8···O10B	0.82	1.91	2.587 (3)	139
O10A—H10A···O9^i^	0.85	2.01	2.844 (3)	168
O10B—H10C···O1^iii^	0.96	2.50	3.357 (5)	148
C11—H11···O9^iv^	0.93	2.44	3.345 (3)	164
C23—H23···Cg1^v^	0.93	2.65	3.562 (2)	168

Symmetry codes: (i) *x*+1, *y*, *z*; (ii) −*x*+1, −*y*+1, −*z*; (iii) −*x*+2, −*y*+1, −*z*; (iv) −*x*, −*y*+1, −*z*+1; (v) −*x*+1, −*y*+1, −*z*+1.

### Table 2 Table 2 π-π stacking interactions (Å,°)

Cg1, Cg2 and Cg3 are the centroids of the C1–C6, C10–C15 and C19–C24 rings, respectively.

**Table:**

CgI	CgJ	CgI···CgJ^a^	CgI···P(J)^b^	CgJ···P(I)^c^	Slippage
Cg1	Cg1^vi^	3.5314 (11)	3.396	3.396	0.968
Cg2	Cg2^vii^	3.6525 (14)	3.377	3.377	1.392
Cg3	Cg3^v^	3.7905 (14)	3.358	3.358	1.758

Symmetry codes: (v) 1-x, 1-y, 1-z; (vi) 1-x, 1-y, -z; (vii) -x, -y, 1-z Notes: (a) Distance between centroids; (b) perpendicular distance of CgI on ring plan J; (c) perpendicular distance of CgJ on ring plan I; (d) slippage = vertical displacement between ring centroids.
